# Retraction: He-Wei granules (HWKL) combat cisplatin-induced nephrotoxicity and myelosuppression in rats by inhibiting oxidative stress, inflammatory cytokines and apoptosis

**DOI:** 10.1039/d3ra90036c

**Published:** 2023-04-19

**Authors:** Zehai Song, Hang Chang, Na Han, Zhihui Liu, Ye Liu, Hui Wang, Jingxuan Shao, Zhonglin Wang, Hao Gao, Jun Yin

**Affiliations:** a Development and Utilization Key Laboratory of Northeast Plant Materials, School of Traditional Chinese Materia Medica 48#, Shenyang Pharmaceutical University Wenhua Road 103, Shenhe District Shenyang 110016 China yinjun2002@yahoo.com +86-24-2398-6460 +86-24-2398-6491; b Beijing Handian Pharmaceutical Co., Ltd Beijing 100020 China

## Abstract

Retraction of ‘He-Wei granules (HWKL) combat cisplatin-induced nephrotoxicity and myelosuppression in rats by inhibiting oxidative stress, inflammatory cytokines and apoptosis’ by Zehai Song *et al.*, *RSC Adv.*, 2017, **7**, 19794–19807, https://doi.org/10.1039/C7RA02830J.

The Royal Society of Chemistry, with the agreement of the authors, hereby wholly retracts this *RSC Advances* article due to concerns with the reliability of the data.

Concerns were initially raised with the integrity of the western blots in Fig. 6.

The authors provided raw data for the western blot images which does not appear to be genuine. The raw data shows signs of cloning as duplicating features can be observed, which indicates that the images have been manipulated.

The authors state that they outsourced the western blot experiments in this paper and have offered to re-perform this work. However, given the significance of the concerns about the validity of both the data in the article and the raw data provided by the authors, the findings presented in this paper are not reliable.

Signed: Zehai Song, Hang Chang, Na Han, Zhihui Liu, Ye Liu, Hui Wang, Jingxuan Shao, Zhonglin Wang, Hao Gao and Jun Yin

Date: 24th March 2023

## Supplementary Material

